# Transcription of [FeFe]-Hydrogenase Genes during H_2_ Production in *Clostridium* and *Desulfovibrio* spp. Isolated from a Paddy Field Soil

**DOI:** 10.1264/jsme2.ME16171

**Published:** 2017-05-13

**Authors:** Ryuko Baba, Mayumi Morita, Susumu Asakawa, Takeshi Watanabe

**Affiliations:** 1 Laboratory of Soil Biology and Chemistry, Department of Biological Mechanisms and Functions, Graduate School of Bioagricultural Sciences, Nagoya UniversityFurocho, Chikusa, Nagoya 464–8601Japan; 2 Laboratory of Soil Biology and Chemistry, School of Agricultural Sciences, Nagoya UniversityFurocho, Chikusa, Nagoya 464–8601Japan

**Keywords:** paddy field soil, [FeFe]-hydrogenase, H_2_-producing microorganism, *hydA*-paralog, transcriptional analysis

## Abstract

Changes in the relative abundances of the transcripts of *hydA* gene paralogs for [FeFe]-hydrogenase in *Clostridium* sp. strain H2 and *Desulfovibrio* sp. strain A1 isolated from paddy field soil were analyzed during H_2_ production. Strains H2 and A1 had at least five and two phylogenetically different *hydA* genes, respectively. The relative abundances of their *hydA* transcripts differed among the paralogs and H_2_ production activity changed in a manner that depended on the growth phase and conditions. Increases or decreases in the relative abundances of the transcripts of two out of five *hydA* genes in strain H2 correlated with changes in H_2_ production rates, whereas those of the others remained unchanged or decreased. In strain A1, the relative abundances of the transcripts of two *hydA* genes differed between monoculture, sulfate-reducing, and syntrophic, methanogenic conditions. The relative abundance of the transcripts of one *hydA* gene, predicted to encode a cytosolic [FeFe]-hydrogenase, was higher under syntrophic, methanogenic conditions than sulfate-reducing conditions, while that of the transcripts of the other *hydA* gene decreased with time under both conditions. This study showed that the transcription of the *hydA* gene during growth with active H_2_ production was differently regulated among the paralogs in H_2_ producers isolated from paddy field soil.

Molecular H_2_ produced during the anaerobic decomposition of organic matter is one of the important intermediates in anoxic paddy field soil (7, 12). H_2_ is produced by various fermenters using protons as the electron acceptor, and is consumed by H_2_ scavengers such as sulfate reducers and methanogens (32). Although apparent H_2_ production in paddy field soil is very low because of concomitant H_2_ consumption (12), the balance between the production and consumption of H_2_ regulates the decomposition processes of organic matter (7). Thus, elucidating the ecophysiology of key H_2_ producers is crucial for obtaining a more complete understanding of the biogeochemical cycle in paddy field soil.

However, clarification of the diversity, activities, and roles of H_2_ producers in the environment is challenging because H_2_ producers are physiologically and phylogenetically diverse microorganisms. Previous studies estimated the contribution of acetate and CO_2_/H_2_ to methane production and emission from paddy field soils using a stable carbon isotopic signature and tracer experiments (9, 28, 34). The findings obtained showed the importance of H_2_ in methanogenesis in paddy field soil. However, H_2_ producers in paddy field soil have not been examined in detail. A few studies have investigated members that produce H_2_ as secondary fermenters using a stable isotope probing technique with ^13^C-labeled propionate and butyrate in paddy field soil (16, 17).

Hydrogenases are enzymes that catalyze H_2_ metabolism. They are grouped into [NiFe]-, [Fe]-, and [FeFe]-hydrogenases based on the (di)atomic composition of their active sites. [FeFe]-hydrogenases are distributed in anaerobic *Eukarya* and *Bacteria*, which mainly catalyze the production of H_2_ during fermentation; however, certain [NiFe]-hydrogenases also catalyze H_2_ production from formate (38). [FeFe]-hydrogenases exist as monomeric or polymeric FeS proteins, and contain a region called the H cluster encoded by the *hydA* gene (38). We previously conducted a molecular biological analysis targeting *hydA* genes and transcripts in order to examine the diversity of H_2_ producers in paddy field soil (1) and active members during anaerobic rice straw decomposition (2). The findings obtained suggested that *Deltaproteobacteria* and *Firmicutes* were key H_2_ producers in paddy field soil. However, although transcriptional levels of *hydA* in some *Clostridium* species were shown to correlate with the H_2_ production rate (21, 40), the relationship between H_2_ production and the relative abundance of the transcripts of each *hydA* gene remains unknown in paddy field soil because actual H_2_ production activity cannot be evaluated. Moreover, H_2_ producers often possess more than one paralog for [FeFe]-hydrogenases (4, 20, 26), and their functions may differ. For example, *Clostridium* spp. are common H_2_-producing fermenters that have various types of [FeFe]-hydrogenases (4). The transcriptional regulation of the respective *hydA* genes was found to differ during H_2_ production in some *Clostridium* strains isolated from a digested sludge enrichment (22). Many species of *Desulfovibrio* have both periplasmic and cytosolic [FeFe]-hydrogenases (25). *Desulfovibrio* spp., which are representative sulfate-reducing bacteria in anoxic environments, establish a syntrophic relationship with hydrogenotrophic methanogens as secondary fermenters under sulfate-limited conditions (32). Therefore, the two types of [FeFe]-hydrogenases are predicted to play different roles under sulfate-reducing and syntrophic conditions, and their transcriptional patterns may also differ depending on the conditions present. However, information on how H_2_ producers regulate the transcription of *hydA* paralogs during H_2_ production is limited to some defined species (22).

In the present study, we attempted to reveal the transcriptional patterns of *hydA* paralogs during H_2_ production for *Clostridium* sp. strain H2 and *Desulfovibrio* sp. strain A1, which belong to *Firmicutes* and *Deltaproteobacteria*, respectively, isolated from paddy field soil. Both isolates had multiple *hydA* genes in their genomes, and their transcriptional patterns and H_2_-producing activities were analyzed.

## Materials and Methods

### Microorganisms

*Clostridium* sp. strain H2, *Desulfovibrio* sp. strain A1 (NBRC 101757), and *Methanobacterium* sp. strain AH1 (NBRC 103406), which were isolated from paddy field soil in the Aichi-ken Anjo Research and Extension Center, Anjo, Aichi, Japan (Anjo field; latitude 34°58′21″N, longitude 137°04′35″E), were used. The procedures used for isolating and clarifying the physiology and phylogeny of the isolates were described in Supporting information. The sequences of the 16S rRNA genes of strain H2 (LC194786), A1 (AB252583), and AH1 (AB302950 and AB302951) were almost identical (100%, 99% and 99%) to *C. bifermentans* ATCC 638^T^ (AVNC01000016), *D. vulgaris* strain Hildenborough^T^ (AE017285), and *M. palustre* DSM 3108^T^ (AF093061), respectively.

### Sequencing of hydA paralogs in *Clostridium* sp. H2 and *Desulfovibrio* sp. A1

The sequences of the *hydA* genes in the genomes of strains H2 and A1 were elucidated by a PCR-based analysis from the genome information of reference bacteria, *C. bifermentans* ATCC 638 (AVNC00000000), ATCC 19299 (AVNB00000000), and *D. vulgaris* Hildenborough (AE017285). The primer sets targeting each *hydA* gene were designed using Primer3Plus (36) (Table S1). Each *hydA* gene in the genomes of strains H2 and A1 was retrieved by PCR. Each reaction premix (25 μL) contained 2.5 μL of 10×PCR Buffer for KOD-Plus-(Toyobo, Osaka, Japan), 2.5 μL of dNTPs (Toyobo), 1 μL of 25 mM MgSO_4_, 0.5 μL of KOD-Plus-(Toyobo), 0.15 μL of 50 μM forward and reverse primers, and 2.5 μL of template DNA. PCR was performed under the following conditions: 94°C for 2 min, 30 (*H2hydA1*, *H2hydA2*, *H2hydA5*, *A1hydA1*, and *A1hydA2*) or 40 (*H2hydA3* and *H2hydA4*) cycles of 94°C for 15 s, 46°C (*H2hydA3* and *H2hydA4*), 56°C (*H2hydA1*, *H2hydA2* and *H2hydA5*), or 65°C (*A1hydA1* and *A1hydA2*) for 30 s, and 68°C for 2 min. Amplicons were checked by agarose gel electrophoresis followed by ethidium bromide staining. PCR products were purified with the NucleoSpin^®^ Gel and PCR Clean-up kit (Macherey-Nagel, Düren, Germany). A sequencing analysis of each amplicon was performed as described in a previous study (2) after direct cycle-sequencing for *hydA* amplicons using the BigDye^®^ Terminator v3.1 Cycle Sequencing Kit (Applied Biosystems, CA, USA). The nucleotide sequences of the *hydA* genes were translated into amino acid sequences using the EMBOSS Transeq program (EMBL-EBI [http://www.ebi.ac.uk/Tools/st/emboss_transeq/]). A phylogenetic tree was constructed with the H-cluster (38), which contains the conserved active sites of [FeFe]-hydrogenases, of the obtained amino acid sequences and reference sequences by the neighbor-joining method with ClustalW 2.1 on the DDBJ website under default parameters. The tree was formatted using MEGA 5.2 (35). [FeFe]-hydrogenase-like Narf protein sequences (accession no. P23503, Q6CGR3, Q8SYS7) were used as outgroup sequences.

### Preparation of media and incubation of isolates

Three incubation conditions were examined in this study: strain H2 under fermentation conditions, strain A1 under sulfate-reducing conditions, and strains A1 and AH1 under syntrophic, methanogenic conditions. All incubations were performed using Widdel’s freshwater medium (8, 42) with some modifications: medium contained 0.1 g L^−1^ Bacto Yeast Extract and Bacto Peptone (Difco), and 1 mL L^−1^ Wolfe’s vitamin solution (ATCC MD-VS) was used instead of the original vitamin solutions. Sodium sulfate (final concentration, 28 μmol mL^−1^) was added to the medium for strain A1 under sulfate-reducing conditions. Each 200 mL of medium was anoxically prepared in 1-L serum bottles using the Hungate technique (3, 10, 13), and the bottle was closed with a butyl rubber stopper and sealed with an aluminum cap. Triplicate bottles were prepared for each culture condition. The headspace was replaced with filtered N_2_/CO_2_ (the mixing ratio was 4:1) after autoclaving at 121°C for 20 min. Glucose (strain H2) or sodium lactate (monoculture of strain A1 and co-culture of strains A1 and AH1) were added at final concentrations of 44 or 87 μmol mL^−1^, respectively, through a 0.22-μm sterile syringe filter (DISMIC-25AS, Advantec, Tokyo, Japan). Two milliliters of cultures after the third passage under the same culture conditions were inoculated, mixed thoroughly, and incubated under static conditions at 30°C. The co-culture of strains A1 and AH1 was accidentally incubated at room temperature (20–25°C) from 137 h to 164 h after the inoculation.

### Monitoring growth, substrates, and products

Growth was monitored turbidimetrically at 660 nm using a spectrophotometer (UV-2450, Shimadzu, Kyoto, Japan). One milliliter of samples was taken periodically and filtrated using a 0.22-μm syringe filter (DISMIC-25AS, Advantec, Tokyo, Japan) to measure the concentrations of the metabolites and substrates. Filtrates were stored at 4°C until measurements, deproteinized using a pretreatment column Toyopak ODS (Toyobo), and refiltrated with a 0.22-μm syringe filter (DISMIC-25AS, Advantec) just before measurements. Glucose, lactate, formate, and acetate concentrations were measured using a high-pressure liquid chromatograph LC-10AT (Shimadzu) equipped with SUGAR SH1821 (Showa Denko, Tokyo, Japan) with the UV detector SPD-10A (Shimadzu) (210 nm) and differential refractometer R401 (Waters Associates, Manifold, MA, USA). The mobile phase was 0.5 mM sulfuric acid and flowed at a rate of 1.0 mL min^−1^ at 50°C (column temperature). The concentration of sulfate in the culture of strain A1 was measured using the ion chromatograph PIA-1000 (Shimadzu) equipped with TSKgel IC-Anion-PW (Tosoh, Tokyo, Japan) and a conductivity detector. TSKgel eluent IC-Anion-A (Tosoh) was used as a mobile phase and flowed at a rate of 1.0 mL min^−1^ at 35°C (column temperature). The production of H_2_ and methane was measured by gas chromatography, as described by Baba *et al.* (2). H_2_ production rates were calculated from the measured values by the Gompertz modified equation (14) and differential equation. The parameters of the equation were estimated by the Solver function of Microsoft Excel in order to minimize the residual sum of the square between the experiment and estimation.

### RNA extraction and RT-qPCR

Cells were periodically harvested by centrifugation (15,000×*g*, 4°C, 2 min) from 2 mL of cultures and stored at –80°C until used. RNA extraction from the harvested cells was performed using Nucleospin^®^ RNA (Macherey-Nagel), according to the manufacturer’s procedure. DNase I (Promega, Madison, WI, USA) was used for additional DNA digestion. The complete digestion of DNA in RNA preparations was confirmed by PCR using the bacterial universal primer set 357f/517r (23) in the absence of the reverse transcriptase. cDNA was synthesized from RNA preparations using the PrimeScript^®^ RT reagent Kit (Perfect Real Time) (Takara, Otsu, Japan) with random 6-mer primers according to the manufacturer’s instructions.

The synthesized cDNAs were subjected to a qPCR analysis of *hydA* transcripts and 16S rRNAs. Each reaction premix (25 μL) contained 12.5 μL of SYBR^®^ Premix EX Taq (Perfect Real Time) (Takara), 0.1 μL of 50 μM forward and reverse primers (Table S2), and 2 μL of template cDNA or standard DNA (duplicate; 10^1^–10^6^ copies μL^−1^ and 10^4^–10^9^ copies μL^−1^ of *hydA* and 16S rRNA gene fragments, respectively, obtained from the genomic DNAs of strains H2 and A1 by PCR using the primer sets in Table S1). qPCR was performed using a Thermal Cycler Dice Real Time System (Takara) under the following conditions: 95°C for 30 s, and 40 cycles of 95°C for 5 s and 65°C for 45 s. Standard curves showed good reaction efficiencies (76–98%) and R^2^ values (>0.98). The numbers of 16S rRNA and *hydA* transcripts were calculated by absolute quantification based on standard curves. Ct values were obtained by the second derivative maximum method. The relative abundance of *hydA* was calculated with the following formula: the number of *hydA* transcripts/the number of 16S rRNAs at each sampling time. 16S rRNA was used as the normalization reference according to previous studies that quantified the relative abundances of the *hydA* transcripts of microorganisms (22, 40). Bartlett’s test and the Tukey-Kramer test were performed based on the relative abundances of *hydA*, using R (version 3.1.1; R Foundation for Statistical Computing [http://www.R-project.org/]). When the homoscedasticity of data was not confirmed, Dunnett’s T3 test was performed using R package ‘DTK’.

### Accession numbers of nucleotide sequences

The nucleotide sequences of *hydA* obtained in this study have been deposited to the DDBJ database under accession numbers LC194779 to LC194785.

## Results

### *hydA* paralogs in strains H2 and A1

**Fig. 1:**
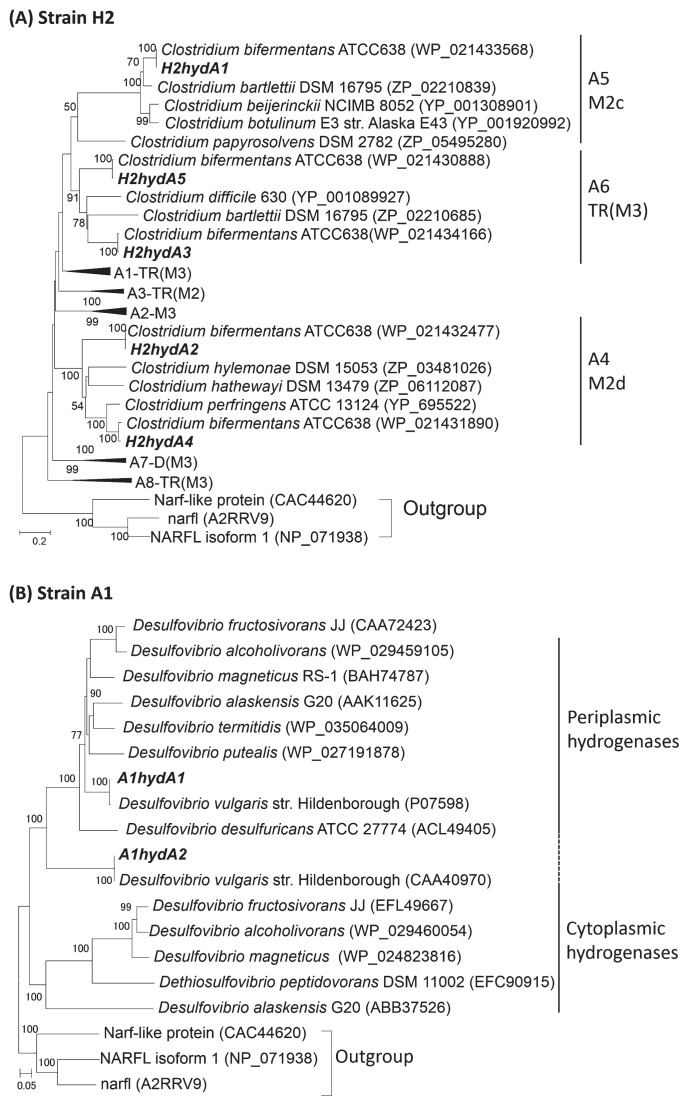
Phylogenetic tree of *hydA* genes possessed by (A) strain H2 and (B) strain A1 (shown in **bold**). The neighbor-joining method was used to make the tree. Bootstrap values (500 resampling, ≥50%) are shown at the nodes. A1–A8 and the names of modular structures (M2, M2c, M2d, TR[M2], M3, DM3, and TR[M3]) are based on the classification of clostridial *hydA* proposed by Calusinska *et al.* (4).

*Clostridium* sp. strain H2 had at least 5 phylogenetically different *hydA* genes (designated as *H2hydA1*–*H2hydA5*) in its genome, and the similarity of these *hydA* genes to the corresponding *hydA* genes of *C. bifermentans* ATCC 638 (AVNC00000000) and ATCC 19299 (AVNB00000000) was very high (99–100%) (Fig. 1A). *Desulfovibrio* sp. strain A1 had at least 2 *hydA* genes (designated as *A1hydA1* and *A1hydA2*, Fig. 1B), and the similarity of these *hydA* genes to the corresponding *hydA* genes of *D. vulgaris* Hildenborough (AE017285) was higher than 99%.

### Growth, metabolites, and H_2_ production

**Fig. 2:**
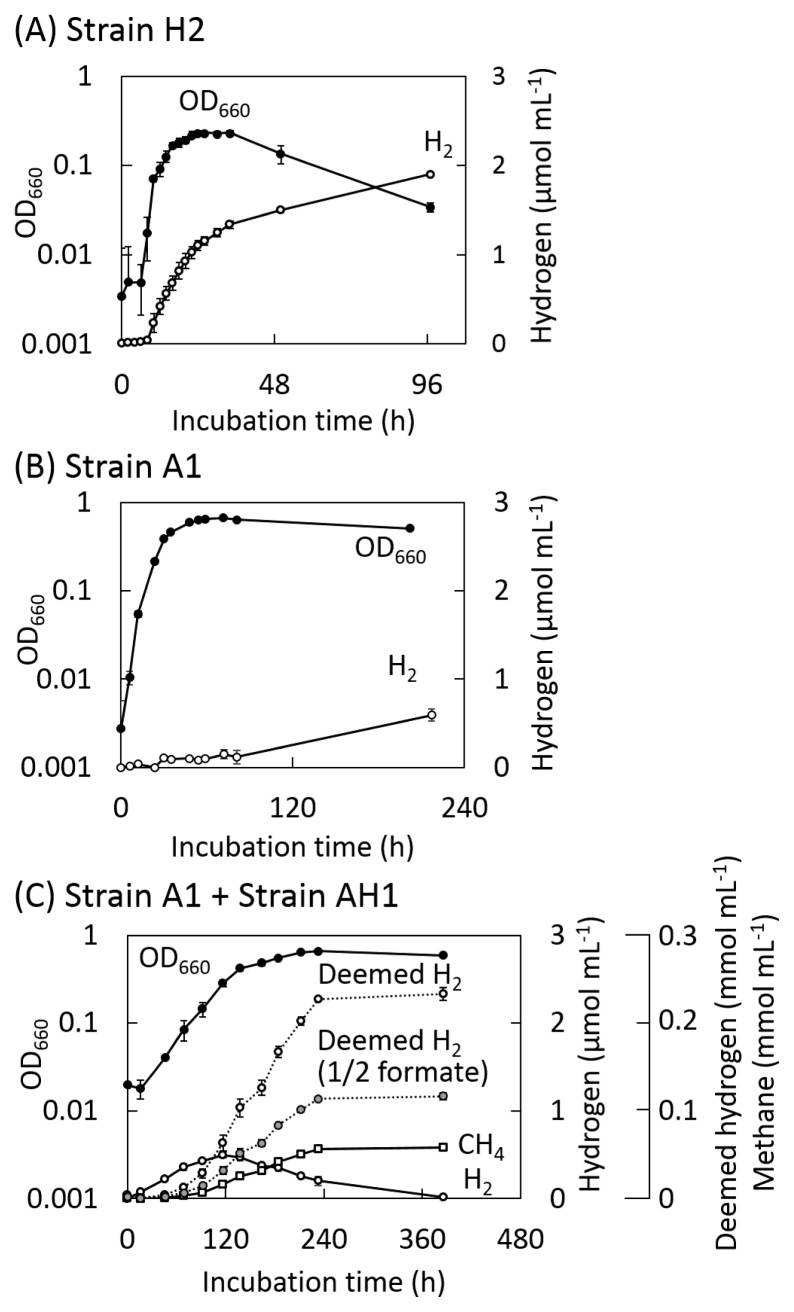
Turbidity and hydrogen and methane production in monocultures of (A) strain H2 and (B) strain A1, and (C) a co-culture of strains A1 and AH1. (bars=S.D., *n*=3). Deemed H_2_ is calculated as the sum of the amount of H_2_ and four-fold of methane. Deemed H_2_ (1/2 formate) is estimated under the assumption when 50% of methane was produced from formate.

**Fig. 3:**
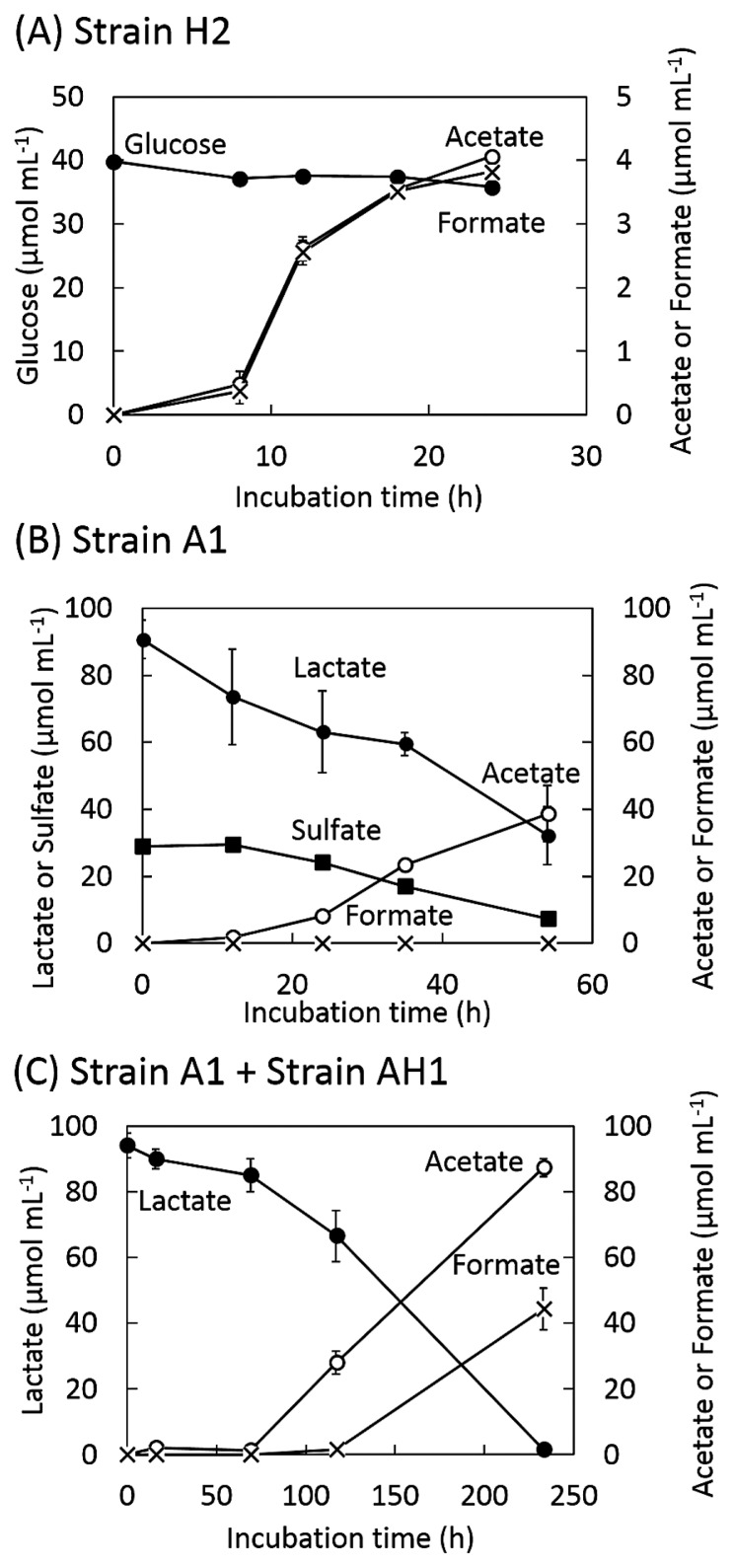
Amounts of substrates (glucose, lactate, and sulfate) and major metabolites in monocultures of (A) strain H2 and (B) strain A1, and (C) a co-culture of strains A1 and AH1. (bars=S.D., *n*=3)

Strain H2 actively grew from 6 to 24 h after the short lag phase (Fig. 2A), and the turbidity of the culture decreased after the stationary phase. Active H_2_ production occurred between 8 and 12 h after the inoculation, and approximately 1.9 μmol mL^−1^ of H_2_ was produced during the 96-h incubation (Fig. 2A). During the active growth phase, the concentration of glucose decreased from 40 μmol mL^−1^ to 36 μmol mL^−1^ and the concentrations of acetate and formate increased to 4.1 and 3.8 μmol mL^−1^ respectively (Fig. 3A).

Strain A1 initiated active proliferation without a lag phase under sulfate-reducing conditions, and growth reached the stationary phase after 30 h (Fig. 2B). Lactate was linearly consumed with the concomitant reduction of sulfate. During growth, the concentration of acetate increased to 39 μmol mL^−1^, while formate was not produced (Fig. 3B). Although H_2_ production was very low during growth, its concentration slightly increased during the stationary phase (Fig. 2B).

In the co-culture of strains A1 and AH1, the turbidity of the culture exponentially increased until 138 h after the short lag phase, and CH_4_ was produced linearly from 70 h to 240 h after the inoculation (Fig. 2C). Lactate was almost consumed during growth, and acetate and formate concentrations increased to 87 and 44 μmol mL^−1^, respectively (Fig. 3C). H_2_ was produced up to 0.50 μmol mL^−1^; however, its concentration decreased with the initiation of CH_4_ production. A total of 0.058 mmol mL^−1^ of CH_4_ was produced at the end of the incubation. Regardless of the assumption that CH_4_ was produced from only H_2_/CO_2_ or both H_2_/CO_2_ and formate (29), H_2_ production continuously occurred during growth (Fig. 2C).

### Relative abundance of transcripts of *hydA* and 16S rRNA genes

Based on H_2_ production activity (Fig. 2), RT-qPCR analyses targeting 16S rRNA and *hydA* transcripts were performed on samples collected at 8, 12, 18, and 24 h for strain H2, 12, 24, 35, and 54 h for strain A1 under sulfate-reducing conditions, and 16, 69, 117, and 233 h after the inoculation for A1 and AH1 under syntrophic, methanogenic conditions.

**Fig. 4:**
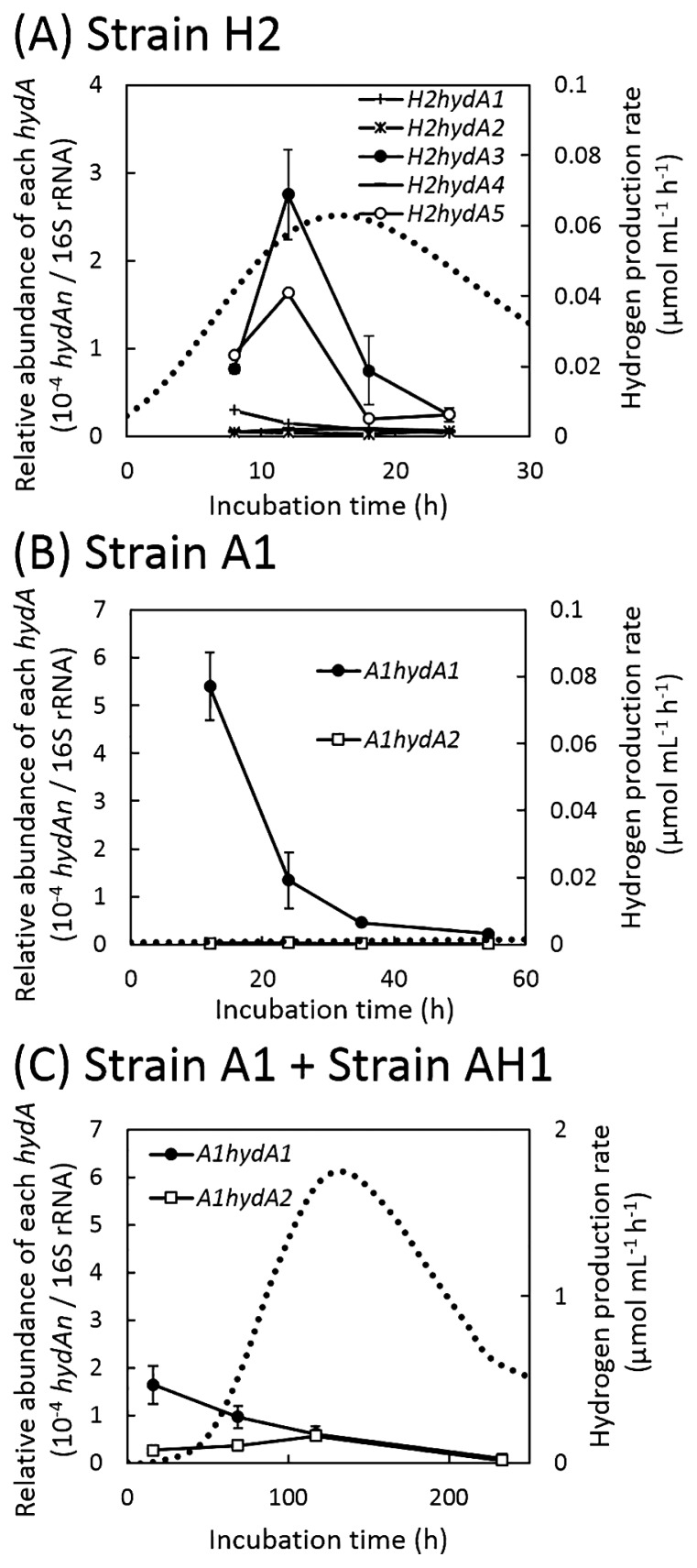
Relative abundance of transcripts of each *hydA* to 16S rRNAs of strains H2 and A1 in monocultures of (A) strain H2, (B) strain A1, and a co-culture of (C) strains A1 and AH1, and the H_2_ production rate (broken lines) calculated using the Gompertz modified equation. The H_2_ production rate of the co-culture (C) was estimated from deemed H_2_ production. (bars=S.D., *n*=3)

In strain H2, the copy numbers of 16S rRNA and *hydA* transcripts were 10^9^–10^10^ copies mL^−1^ and 10^3^–10^6^ copies mL^−1^, respectively, during the incubation. The relative abundances of the transcripts of *H2hydA3* and *H2hydA5* increased 12 h after the inoculation along with increases in the H_2_ production rate; however, the increase observed in the abundance of *H2hydA5* was not significant (*p*=0.12) (Fig. 4A and S7A). The other paralogs (*H2hydA1*, *H2hydA2*, and *H2hydA4*) were also transcribed, but their relative abundances were low, unchanged, or decreased (18 h and 24 h versus 8 h in *H2hydA1*) during H_2_ production (Fig. 4A and S7A).

In strain A1 under sulfate-reducing conditions, the copy numbers of 16S rRNA and *hydA* transcripts were 10^10^–10^11^ copies mL^−1^ and 10^5^–10^7^ copies mL^−1^, respectively. The relative abundances of the transcripts of *A1hydA1* were markedly lower than those at 12 h, while that of *A1hydA2* was always low during growth (Fig. 4B and S7B).

In the co-culture of strains A1 and AH1 under syntrophic, methanogenic conditions, the copy numbers of the 16S rRNA and *hydA* transcripts of strain A1 were 10^8^–10^10^ copies mL^−1^ and 10^3^–10^6^ copies mL^−1^, respectively. Similar to sulfate-reducing conditions, the relative abundances of the transcripts of *A1hydA1* linearly decreased during proliferation (*p*<0.05), whereas those of *A1hydA2* increased until 117 h and then decreased at 233 h after the inoculation, which corresponded to the increase in the deemed H_2_ production rate (Fig. 4C and S7C).

## Discussion

We herein examined the transcriptional regulation of the *hydA* paralogs of two H_2_ producers during H_2_ production and their predicted functions. We also discussed further prospects for elucidating the ecology of H_2_ producers in paddy field soil by a molecular biological analysis targeting *hydA* paralogs.

Strains H2 and A1 had at least 5 and 2 *hydA* paralogs in their genomes by a PCR-based analysis based on the genomic information of their close relatives. The number of paralogs was within a predictable range of *hydA* paralogs in *Clostridium* spp. (2–7 *hydA* paralogs) (4) and *Desulfovibrio* spp. (1–5 *hydA* paralogs) (25). The closest relatives of each *hydA* paralog in strains H2 and A1 were those in *C. bifermentans* ATCC 636 and *D. vulgaris* strain Hildenborough with high similarities (99–100%), which have 5 and 2 *hydA* paralogs in their genomes, respectively. The phylogeny of these *hydA* paralogs was diverse (Fig. 1A), suggesting that strain H2 has multiple [FeFe]-hydrogenases with different modular structures because [FeFe]-hydrogenases with different modular structures are predicted to contain different HydA subunits (4). According to the classification of clostridial *hydA* genes by Calusinska *et al.* (4), the sequence information of the *hydA* paralogs in strain H2 suggested that *H2hydA1*, *H2hydA2*, and *H2hydA4* each encodes the monomeric [FeFe]-hydrogenase, and *H2hydA3* and *H2hydA5* each encodes a catalytic subunit of the trimeric [FeFe]-hydrogenase. The variety of [FeFe]-hydrogenases in strain H2 indicates an interaction with various electron donors because of different numbers of FeS clusters and modules in their structures (4, 38), suggesting the versatile ability of strain H2.

Positive relationships between *hydA* transcription and H_2_ production have previously been reported for *Clostridium* spp. (6, 40). However, the present study showed that the transcriptional regulation of *hydA* in strain H2 differed among the paralogs. Morra *et al.* (22) and Calusinska *et al.* (5) also showed different regulation patterns for *hydA* paralogs in three *Clostridium* species (*C. beijerinckii*, *C. butyricum*, and *C. perfringens*) and *C. butyricum* CWBI 1009. These findings suggested that each *hydA* has different roles in H_2_ metabolism. However, even if a *hydA* paralog has a similar domain structure among different microorganisms, regulating the transcription of the *hydA* paralog may differ depending on the microorganisms. In this study, the relative abundances of the transcripts of *H2hydA3* and *H2hydA5* increased during active H_2_ production (Fig. 4). The transcription of *hydA* paralogs Cbei_4110 (*C. beijerinckii* SM10; [22]) and CBY_2047 (*C. butyricum* SM32; [22]), which had a TR(M3) structure (22), was constant during H_2_-producing growth. However, the relative abundances of the transcripts of *H2hydA3* and *H2hydA5* of strain H2, which were phylogenetically grouped into A6-TR(M3) [FeFe]-hydrogenases (Fig. 1A), changed during H_2_-producing growth. Therefore, the regulation of *hydA* transcription during H_2_ production may depend not only on the types of *hydA*, but also on microorganisms and growth (environmental) conditions.

*H2hydA3* in strain H2 was phylogenetically close to *hydA* genes encoding a subunit of bifurcating [FeFe]-hydrogenases, which produce H_2_ by receiving electrons from not only reduced ferredoxin, but also NADH (31). Bifurcating [FeFe]-hydrogenases may catalyze H_2_ production under low H_2_ pressure (31, 33, 43) possibly when the amount of ATP synthesized increases in cells (43). Therefore, this bifurcating [FeFe]-hydrogenase partly encoded in *H2hydA3* appears to contribute to H_2_ production when substrates are rich and H_2_ consumers co-exist.

Two *hydA* paralogs possessed by strain A1 also differed with each other in terms of their phylogeny, although both were closely related to the *hydA* genes of *D. vulgaris* strain Hildenborough. *A1hydA1* and *A1hydA2* were predicted to encode the periplasmic [FeFe]-hydrogenase and cytosolic bifurcating [FeFe]-hydrogenase, respectively, according to the genomic analysis of sulfate reducers (25, 37).

*A1hydA1* and *A1hydA2* were both transcribed under sulfate-reducing and syntrophic conditions (Fig. 4B and C). Active H_2_ production was not observed under sulfate-reducing conditions; however, the relative abundance of the transcripts of *A1hydA1* was high at the initial growth phase (Fig. 4B). Since periplasmic [FeFe]-hydrogenases in *Desulfovibrio* species are known to catalyze H_2_ consumption (25, 27, 38), H_2_ produced by strain A1 may be consumed in the sulfate-reducing process in parallel, as indicated by Odom and Peck (24). However, since the relative abundance of the transcripts of the other *hydA* paralog (*A1hydA2*) did not change under sulfate-reducing conditions, the role of *A1hydA1* in H_2_ metabolism currently remains unknown.

Under syntrophic conditions, active H_2_ production occurred as CH_4_ was actively produced by the hydrogenotrophic methanogenic archaeon strain AH1; however, formate also appears to be utilized in part for CH_4_ production. Under these conditions, the transcriptional pattern of *A1hydA1* was similar to that under sulfate-reducing conditions, namely, the relative abundance of the transcripts decreased with time. On the other hand, the relative abundance of the transcripts of *A1hydA2* was 6–31-fold higher than that under sulfate-reducing conditions, particularly at the time point of active H_2_ production (Fig. 4C), suggesting that *A1hydA2* of strain A1 is related to H_2_ production in syntrophic methanogenesis. As described above, *A1hydA2* is predicted to encode a cytosolic bifurcating [FeFe]-hydrogenase (25, 37). However, the role of bifurcating [FeFe]-hydrogenases in sulfate reducers including *Desulfovibrio* species in H_2_ metabolism remains unclear (25), and, thus, further studies are needed.

The periplasmic [FeFe]-hydrogenases of sulfate reducers have been reported to play an important role in interspecies H_2_ transfer. For example, *D. vulgaris* strain Hildenborough, which has a mutation in the gene of periplasmic [FeFe]-hydrogenase, showed a low growth rate under sulfate-deficient syntrophic conditions co-cultured with a hydrogenotrophic methanogen (39). *D. alaskensis* G20 increased the relative abundances of the transcripts of the gene of periplasmic [FeFe]-hydrogenase under syntrophic conditions (15). On the other hand, the relative abundance of the transcripts of the gene of *D. alaskensis* G20 did not differ under sulfate-reducing and syntrophic conditions, and periplasmic [NiFe]-hydrogenases and formate dehydrogenases may contribute to interspecies electron transfer (18). Syntrophic partners (H_2_ scavengers) also influenced the relative abundance of the transcripts of the hydrogenase genes of H_2_ producers (19). Discrepancies between these findings and the present results suggest that the roles of periplasmic [FeFe]-hydrogenases in H_2_ metabolism differ depending on not only the strains, but also growth conditions. Strain A1 may have independently developed a unique H_2_ metabolism system to adapt to the environment of paddy field soil, thereby influencing the transcriptional patterns of *hydA* genes.

Many bacteria have multiple [FeFe]-hydrogenase genes in their genomes (4, 25, 30, 37, 38). This study showed that the relative abundances of the transcripts of some *hydA* did not have positive relationships with H_2_ production irrespective of high or low relative abundances. These findings indicate that analyses of *hydA* in the environment are associated with the risk of overestimating the diversity and activity of potential H_2_ producers, as already discussed in other studies (1, 30). Meanwhile, this study showed the up-regulated transcription of some *hydA* (*H2hydA3*, *H2hydA5*, and *A1hydA2*) during H_2_ production and differences in the relative abundances of the transcripts between sulfate-reducing and syntrophic conditions. Some *hydA* transcripts (14R348 [LC041901] and 1dR151 [LC041552] in Fig. S8) closely related to *H2hydA3* and *H2hydA5* of strain H2 and *A1hydA2* of strain A1 were actually detected during anaerobic rice straw decomposition in paddy field soil (2). These findings indicate that the transcription of some *hydA* certainly reflects active H_2_ production.

Paddy fields, in which apparent H_2_ production is very low (12), are flooded during rice cultivation, and the field is drained after rice harvest, resulting in soil conditions that markedly change between oxic and anoxic conditions. These changes affect the metabolic activities and growth/survival strategies of microorganisms to adapt to these changes; however, the community structures of bacteria and methanogenic archaea are known to be stable irrespective of dynamic changes in soil conditions (11, 41). The roles and active members of H_2_ producers must also change and shift dynamically during H_2_ production depending on soil conditions. However, limited information is available on the regulation of H_2_ production by soil microorganisms. Paddy field soil harbors numerous microorganisms including diverse H_2_ producers (1,2). The structure and function of [FeFe]-hydrogenases and the transcriptional regulation of the *hydA* paralogs of soil H_2_ producers need to be diverse. Therefore, further studies on the regulation of the *hydA* paralogs of various soil isolates are needed and will provide new perspectives for understanding active H_2_ producers and H_2_-dependent microbial interactions in paddy field soil.

## Supporting information
