# Supplementary material for: Transcription of [FeFe]-Hydrogenase Genes during H2 Production in Clostridium and Desulfovibrio spp. Isolated from a Paddy Field Soil
Source: Microbes Environ. 2017 May 13;32(2):125–32. doi: 10.1264/jsme2.ME16171 (PMC5478535; doi:10.1264/jsme2.ME16171)
Supplement: Supplementary file 1 [file 32_125_s1.pdf]

Supporting information

Transcription of [FeFe]-hydrogenase genes during H_2 production in *Clostridium* and *Desulfovibrio* spp. isolated from a paddy field soil.

Ryuko Baba, Mayumi Morita, Susumu Asakawa, Takeshi Watanabe

Isolation of microorganisms

Materials and methods

Isolation of Clostridium *sp. strain H2*

A moist soil sample (about 10 g), which was collected from the plowed layer of a paddy field (Anthraquic Yellow Soil; Oxyaquic Dystrudepts) in the Aichi-ken Anjo Research and Extension Center, Anjo, Aichi, Japan (Anjo field; latitude 34°58'21"N, longitude 137°04'35"E) on April 17th, 2012, was used as the source of inoculum. The soil was homogenized with 90 mL of sterilized anoxic water for 15 min by a shaker, and incubated at 80°C for 10 min in a water bath. Two mL of the dilution were inoculated into 20 mL of VL agar medium (18 g L^{-1} Nutrient Broth [Eiken Chemical, Tochigi, Japan]; 5 g L^{-1} Bacto Yeast Extract [Difco, Becton Dickinson, Sparks, USA]; 0.3 g L^{-1} L-cysteine hydrochloride hydrate; 2 g L^{-1} glucose; 1.5 % [w/v] agar) keeping the temperature at 50-60°C. Then the mixture was solidified in an upended, sterilized outer glass petri dish. After 10 mL

of 1.5 % agar-containing 50 mg L^{-1} L-cysteine hydrochloride hydrate were overlaid to the solidified mixture, bottom face of the sterilized inner glass petri dish was pressed onto the agar to shut off air supply (double-petri dish method: (16)). The petri dish was covered with a plastic wrap and incubated at 30°C for 1 month. Bacterial colonies formed under the bottom face on the medium were picked up by sterilized Pasteur pipettes under filtered N_2 gas atmosphere and inoculated into 5 mL of VL liquid medium in test tubes, which were prepared anoxically by the Hungate technique (6,12,14). The test tube was closed with a butyl rubber cap and a screw cap and incubated at 30°C under N_2 gas phase. The culture after growth was purified by the roll-tube technique, and then well-isolated colonies formed on the agar in the roll-tube were picked up and transferred into a new VL liquid medium. To select H_2 producers, H_2 production activity was monitored by gas chromatography as described in Baba *et al.* (5). This purification cycle was repeated at least three times, and then a pure culture was obtained finally. Purity was confirmed by preparing roll tubes inoculated with serial dilutions of the culture and examining homogeneity of colony morphology, in addition to the homogeneity of cell morphology by microscopic observations. DNA extraction, PCR amplification, cloning and sequencing steps for direct

determination of 16S rRNA gene sequence were described previously (4,13). The primer set of 27f and 1492r (19) was used for the analysis (Table S1). Gram staining was checked by the Hucker method. Cell morphology was observed by a phase contrast microscope (BX50, Olympus, Tokyo, Japan). The pure culture was designated strain H2. Nucleotide sequence of 16S rRNA gene has been deposited to the DDBJ database under the accession number LC194786.

Isolation of* Desulfovibrio *sp. A1 and* Methanobacterium *sp. AH1

A sulfate-reducing bacterium and a methanogenic archaeon were isolated from a methanogenic consortium enriched on acetate from a paddy field soil. The composition of the basal medium used for enrichment and isolation was based on the medium for sulfate reducers (Widdel's defined medium; (8)) originated from the basal freshwater medium (20). The basal medium contained 0.2 g L^{-1} KH_2PO_4, 0.25 g L^{-1} NH_4Cl, 1.0 g NaCl, 0.4 g L^{-1} $MgCl_2·6H_2O$, 0.5g L^{-1} KCl, $CaCl_2·2H_2O$, 1 mL L^{-1} nonchelated trace element mixture, 1 mL L^{-1} selenite-tungstate solution, 1 L^{-1} mL vitamin mixture (8), 1 mL L^{-1} vitamin B_{12} solution (20), 0.5 mL L^{-1} 0.2% (w/v) resazurin, 0.5 g L^{-1} L-cysteine hydrochloride hydrate, 0.5 g L^{-1} $Na_2S·9H_2O$ and 4.8 g L^{-1} $NaHCO_3$. For enrichment, 4.1 g L^{-1} sodium acetate was added to the basal medium. The gas phase was N_2:CO_2 (mixing ratio was 4:1, 203 kPa) and the pH was 7.0. The medium was prepared

anoxically, basically according to the Hungate technique (6,12,14).

Enrichment was performed in a 1 L serum bottle containing 100 mL medium sealed with a butyl rubber stopper and an aluminum seal. A moist soil sample (about 20 g), which was collected from a paddy field plot (Anthraquic Yellow Soil; Oxyaquic Dystrudepts) in the Aichi-ken Anjo Research and Extension Center, Anjo, Aichi, Japan on 28 July 2003 (18), was inoculated into the medium and the bottle was incubated statically at 30˚C. About 90% of the culture was replaced by fresh medium about once per one to two months with monitoring methane production. Methane production was determined by gas chromatography as described previously (2) with a GC-9A gas chromatograph equipped with a frame ionization detector (Shimadzu, Kyoto, Japan). The enrichment culture was scaled down to a 20 mL medium in a 120 mL serum bottle since the fourth transfer. After the following two transfers, roll tubes were prepared. For roll tubes, 1.7% (w/v) agar was added to the medium. The well-isolated colonies were inoculated into 5 mL of liquid medium in test tubes and methane production was followed with time after incubation. Two mL of a culture that formed methane was inoculated into the fresh medium in a 120 mL serum bottle. After two successive transfers as described above, the culture was subjected to

microscopic observation and denaturing gradient gel electrophoresis (DGGE) to examine the archaeal/bacterial communities in the culture. Epifluorescent microscopy was conducted with a BX50 microscope (Olympus, Tokyo, Japan). DGGE analysis of methanogenic archaeal 16S rRNA gene was carried out for the extracted DNA from the culture by bead-beating method with a primer set of 1106F-GC/1378R (17,18). DGGE analysis of bacterial 16S rRNA gene in the culture was conducted for the extracted DNA as described by Asari *et al.* (3).

One mL of the culture was transferred into 10 mL of the basal medium without acetate and incubated under H_2/CO_2 (mixing ratio was 4:1, 203 kPa) atmosphere to isolate a hydrogenotrophic methanogenic archaeon. Roll tubes were prepared for the culture on H_2/CO_2 and well-isolated colonies were picked up and inoculated into 5 mL liquid medium in test tubes. A culture that grew with methane production was subcultured in a 120 mL serum bottle in 10 mL medium. To test for purity, the culture was inoculated into three kinds of media as follows: 1) the basal medium containing 1% (w/v) glucose, 0.1% Bacto Yeast Extract (Difco) and 0.1% Polypepton (Nihon Pharmaceutical, Osaka, Japan), 2) the basal medium containing 30 mM Na_2SO_4 and 20 mM sodium lactate and 3) VL medium under N_2/CO_2 (4:1, 203 kPa) atmosphere for anaerobic heterotrophs as

described previously (1). The preparations were incubated statically for 30 days at 30˚C. No growth was observed and no contaminants were detected microscopically. The pure culture was designated strain AH1.

The basal medium without acetate but containing 8.8 g L^{-1} sodium pyruvate was used to isolate a bacterium from the enrichment culture. Two mL of the enrichment culture were inoculated into 20 mL of the liquid medium and incubated at 30˚C. The medium became turbid within 3 days and proliferation of curved rods was observed in the culture. Roll tubes were then prepared for the culture and well-isolated colonies were picked up to 5 mL liquid medium in the test tubes. A grown culture was subcultured in 20 mL of the basal medium. Isolation of colonies from roll tubes was repeated for purification and well-isolated colonies were inoculated into the liquid medium. Purity was confirmed by preparing roll tubes inoculated with serial dilutions of the culture and examining homogeneity of colony morphology and proportional changes in number of colonies according to dilution. The pure culture was designated strain A1.

DNA extraction, PCR amplification, cloning and sequencing steps for determination of 16S rRNA gene sequences of strains AH1 and A1 were

described previously (13,17). The primers were same as those described by Watanabe *et al*. (17) and 1106F and 1378R (18) were also used for strain AH1. The primers were 27f, 1492r (19), f2L, f3L, r2L, r3L (9,10), rE1L (11) and 926f (15) together with 357f and 517r used for DGGE analysis for strain A1.

Morphology of cells of the isolates was observed by phase contrast and epifluorescent microscopy as described above and also by transmission electron microscopy. Cells for electron microscopy were collected from a late logarithmic culture at 10,000 *g* for 2 min. Resuspended cells were stained negatively with 20 g L^{-1} uranyl acetate after treating with 0.5 % (vol/vol) glutaraldehyde solution and observed with a Hitachi H-7500 AMT Advantage HR electron microscope (Hitachi High-Technology, Tokyo, Japan). Gram staining was carried out by the Hucker method. Substrate utilization of strain A1 was tested in the basal media with and without sodium sulfate (29 mM).

To examine syntrophic methanogenesis of strains AH1 and A1, the strains were cultivated in 5 mL basal media in test tubes or 20 mL basal media in serum bottles containing acetate, pyruvate, lactate, ethanol, malate or fumarate as described above. Growth was measured turbidimetrically at 660 nm and methane production was determined as described above.

Methanobacterium sp. strain AH1 and *Desulfovibrio* sp. strain A1 have been deposited under the numbers of NBRC 103406 and NBRC 101757, respectively, in the culture collection of NITE (National Institute of Technology and Evaluation) Biological Resource Center, Japan. The 16S rRNA gene sequences are available under the accession numbers AB302950 and AB302951 for strain AH1 and AB252583 for strain A1, respectively, in the database of DNA Data Bank of Japan (DDBJ).

Results

Characteristics of strains H2, A1 and AH1

Cell morphology of strain H2 was rod with the size 0.7 – 1.0 × 1.5 – 4.9 μm (Fig. S1). Gram staining was positive. The 16S rRNA gene sequence showed more than 99% similarity with the sequence of *Clostridium bifermentans* ATCC 638^T (Fig. S2).

Cell morphology of strain A1 was curved rod (vibrio) with the size 0.2 – 0.6 × 1.5 – 2.7 μm (Fig. S3). Gram staining was negative. The 16S rRNA gene sequence showed more than 99% similarity with *Desulfovibrio vulgaris* Hildenborough^T (= DSM644^T) (Fig. S4). Strain A1 utilized pyruvate (80 mM), but acetate (50 mM), lactate (30 mM), malate (20 mM) and fumarate (20 mM) were not metabolized. However, when sodium sulfate (29 mM) was added to the basal

medium, strain A1 grew on pyruvate (80 mM), lactate (30 mM) and ethanol (0.2% [v/v]), but not acetate (50 mM).

Cell morphology of strain AH1 was long rod with the size 0.3 - 0.6 μm × 2 - 8 μm (Fig. S5). The 16S rRNA gene sequence showed more than 99% similarity with *Methanobacterium palustre* DSM 3108^T (Fig. S6). Strain AH1 produced methane from H_2/CO_2 and formate. The co-culture of strains AH1 and A1 utilized pyruvate, lactate and ethanol associated with methane production under the condition without sulfate, but not acetate, malate and fumarate.

References

1. Asakawa, S., H. Morii, M. Akagawa-Matsushita, Y. Koga, K. Hayano. 1993. Characterization of *Methanobrevibacter arboriphilus* SA isolated from a paddy field soil and DNA-DNA hybridization among *M. arboriphilus* strains. Int. J. Syst. Evol. Microbiol. 43:683–686 (Erratum [1994] 44, 185).
2. Asakawa, S., K. Hayano. 1995. Populations of methanogenic bacteria in paddy field soil under double cropping conditions (rice-wheat). Biol. Fertil. Soils. 20:113–117.
3. Asari, N., R. Ishihara, Y. Nakajima, M. Kimura, S. Asakawa. 2007. Succession and phylogenetic composition of eubacterial communities in rice

straw during decomposition on the surface of paddy field soil. Soil Sci. Plant Nutr. 53:56–65.

4. Baba, R., M. Kimura, S. Asakawa, T. Watanabe. 2014. Analysis of [FeFe]-hydrogenase genes for the elucidation of a hydrogen-producing bacterial community in paddy field soil. FEMS Microbiol. Lett. 350:249–256.
5. Baba, R., S. Asakawa, T. Watanabe. 2016. H_2-producing bacterial community during rice straw decomposition in paddy field soil: Estimation by an analysis of [FeFe]-hydrogenase gene transcripts. Microbes Environ. 31:226–233.
6. Balch, W., G. Fox, L. Magrum, C. Woese, R. Wolfe. 1979. Methanogens: re-evaluation of a unique biological group. Microbiol. Rev. 43:260–269.
7. DeLong, E. F. 1992. Archaea in coastal marine environments. Proc. Natl. Acad. Sci. U. S. A. 89:5685–5689.
8. Fukui, M. 1992. Enumeration and isolation of sulfate reducers. p. 312–321. In Japanese Society of Soil Microbiology (ed.), Experimental methods for soil microorganisms (Dojobiseibutsujikkensho), Yokendo, Tokyo.
9. Hiraishi, A. 1992. Direct automated sequencing of 16S rDNA amplified by polymerase chain reaction from bacterial cultures without DNA purification.

Lett. Appl. Microbiol. 15:210–213.

10. Hiraishi, A., Y. Shin, Y. Ueda, J. Sugiyama. 1994. Automated sequencing of PCR-amplified 16S rDNA on “Hydrolink” gels. J. Microbiol. Methods. 19:145–154.

11. Hiraishi, A. 1994. Phylogenetic affiliations of *Rhodoferax fermentans* and related species of phototrophic bacteria as determined by automated16S rDNA sequencing. Curr. Microbiol. 28:25–29.

12. Hungate, R. 1969. A roll tube method for cultivation of strict anaerobes. Methods Microbiol. 3B:117–132.

13. Ikenaga, M., S. Asakawa, Y. Muraoka, M. Kimura. 2003. Bacterial communities associated with nodal roots of rice plants along with the growth stages: Estimation by PCR-DGGE and sequence analyses. Soil Sci. Plant Nutr. 49:591–602.

14. Koga, Y., H. Morii, M. Nishihara. 1987. Methods for isolation and cultivation of methanogenic bacteria. Hakkokogaku. 65:419–430.

15. Lane, D., B. Pace, G. Olsen, D. Stahl, M. Sogin, N. Pace. 1985. Rapid determination of 16S ribosomal RNA sequences for phylogenetic analyses. Proc. Natl. Acad. Sci. U. S. A. 82:6955–6959.

16. Takeda, K. 1992. Anaerobic bacteria. p. 23–35. In Japanese Societies of Soil Microbiology (ed.), Experimental methods for soil microorganisms (Dojobiseibutsujikkensho), Yokendo, Tokyo.

17. Watanabe, T., S. Asakawa, A. Nakamura, K. Nagaoka, M. Kimura. 2004. DGGE method for analyzing 16S rDNA of methanogenic archaeal community in paddy field soil. FEMS Microbiol. Lett. 232:153–163.

18. Watanabe, T., M. Kimura, S. Asakawa. 2006. Community structure of methanogenic archaea in paddy field soil under double cropping (rice–wheat). Soil Biol. Biochem. 38:1264–1274.

19. Weisburg, W. G., S. M. Barns, D. a Pelletie, D. J. Lane, D. a. Pelletier, D. J. Lane. 1991. 16S ribosomal DNA amplification for phylogenetic study. J. Bacteriol. 173:697–703.

20. Widdel, F., F. Bak. 1992. Gram-negative mesophilic sulfate-reducing bacteria. p. 3352–3378. In A. Balows, H.G. Trüpe, M. Dworkin, W. Harder, K.-H. Schleifer (eds.), The Prokaryotes, 2nd ed., Springer, New York.

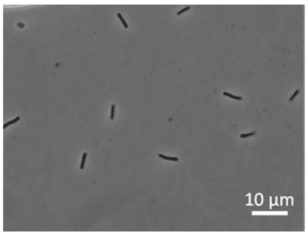

Fig. S1 Phase contrast micrograph of strain H2. Bar = 10 µm.

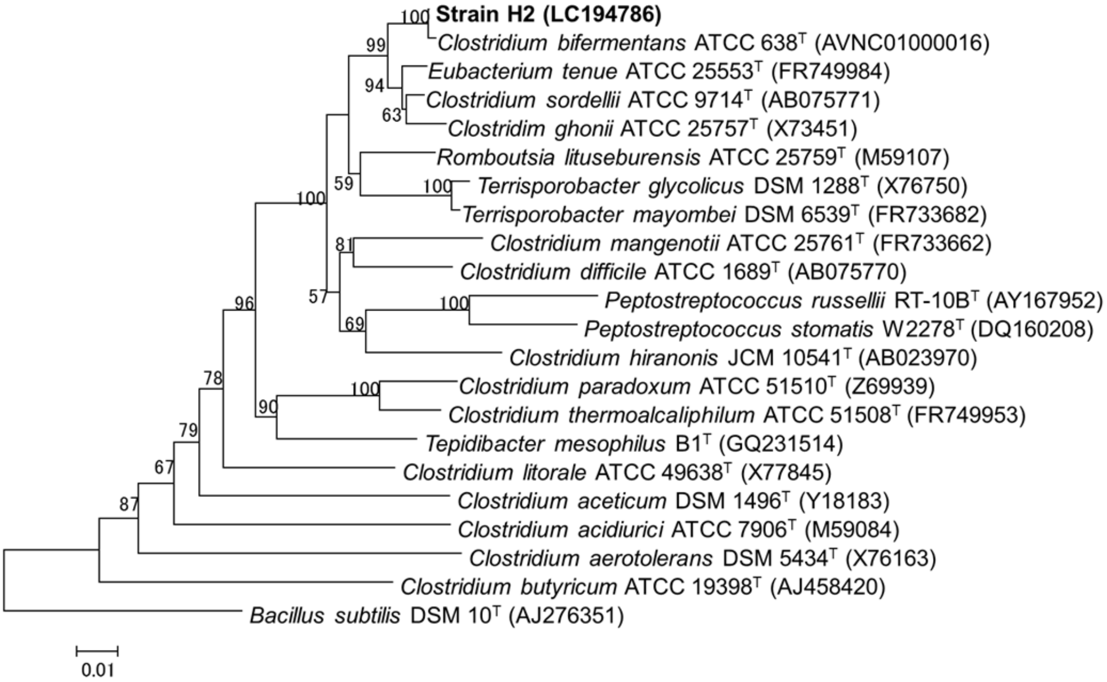

Fig. S2 Phylogenetic tree of 16S rRNA gene possessed by strain H2 (shown in **bold**). Neighbor joining method was used for making the tree. Bootstrap values (1000 resampling) were shown in the nodes.

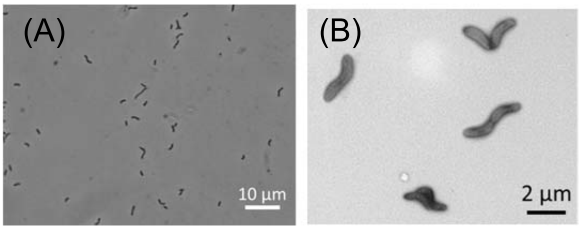

Fig. S3 (A) Phase contrast and (B) transmission electron micrographs of strain A1. Bar = (A) 10 µm and (B) 2 µm.

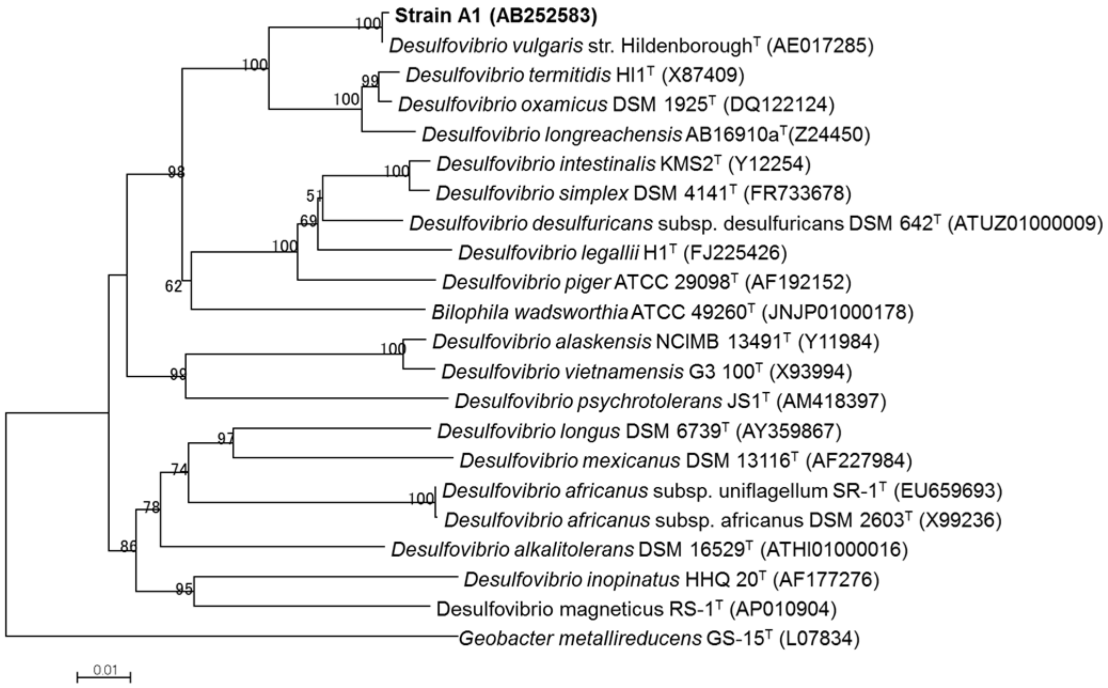

Fig. S4 Phylogenetic relationship of 16S rRNA gene possessed by strain A1 (shown in **bold**). Neighbor joining method was used for making the tree. Bootstrap values (1000 resampling) were shown in the nodes.

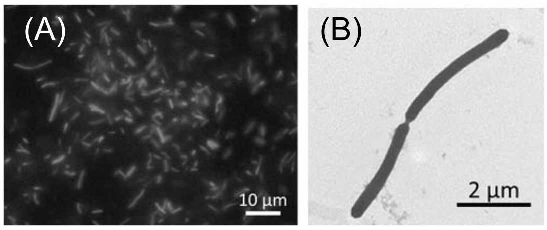

Fig. S5 (A) Autofluorescence and (B) transmission electron micrographs of strain AH1. Bar = (A) 10 µm and (B) 2 µm.

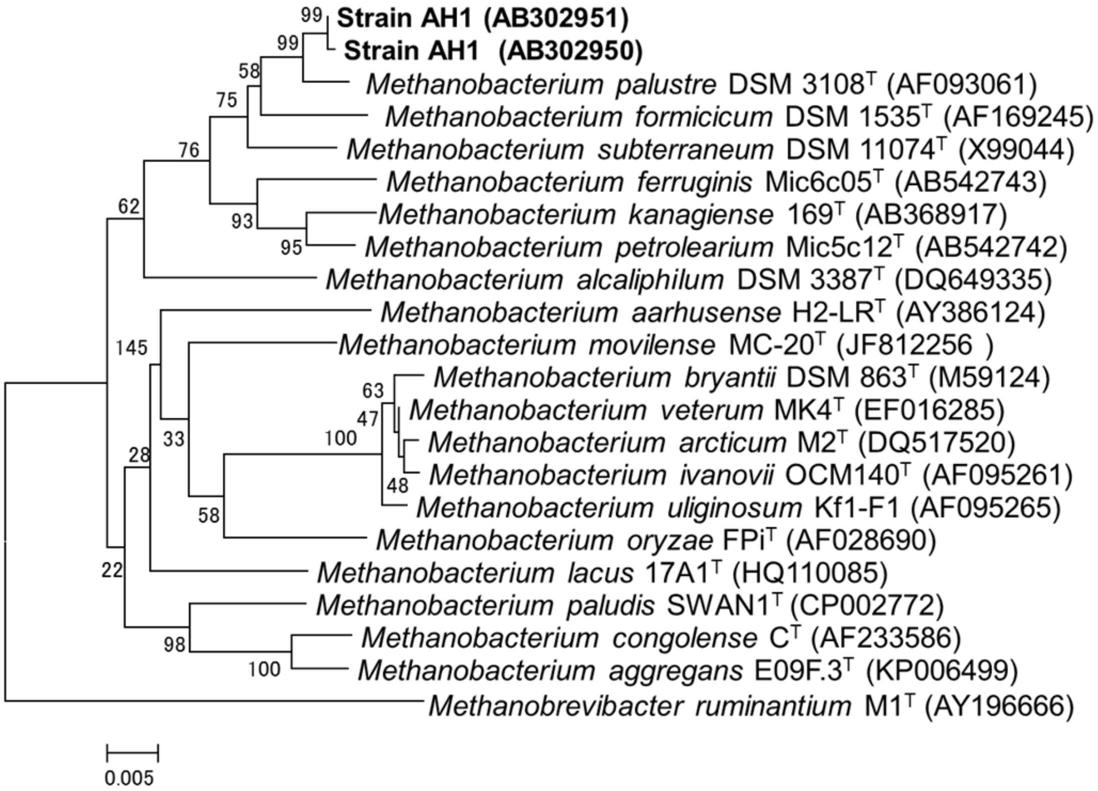

Fig. S6 Phylogenetic relationship of 16S rRNA genes possessed by strain AH1 (shown in **bold**). Neighbor joining method was used for making the tree. Bootstrap values (1000 resampling) were shown in the nodes.

Statistical analysis for the results of RT-qPCR

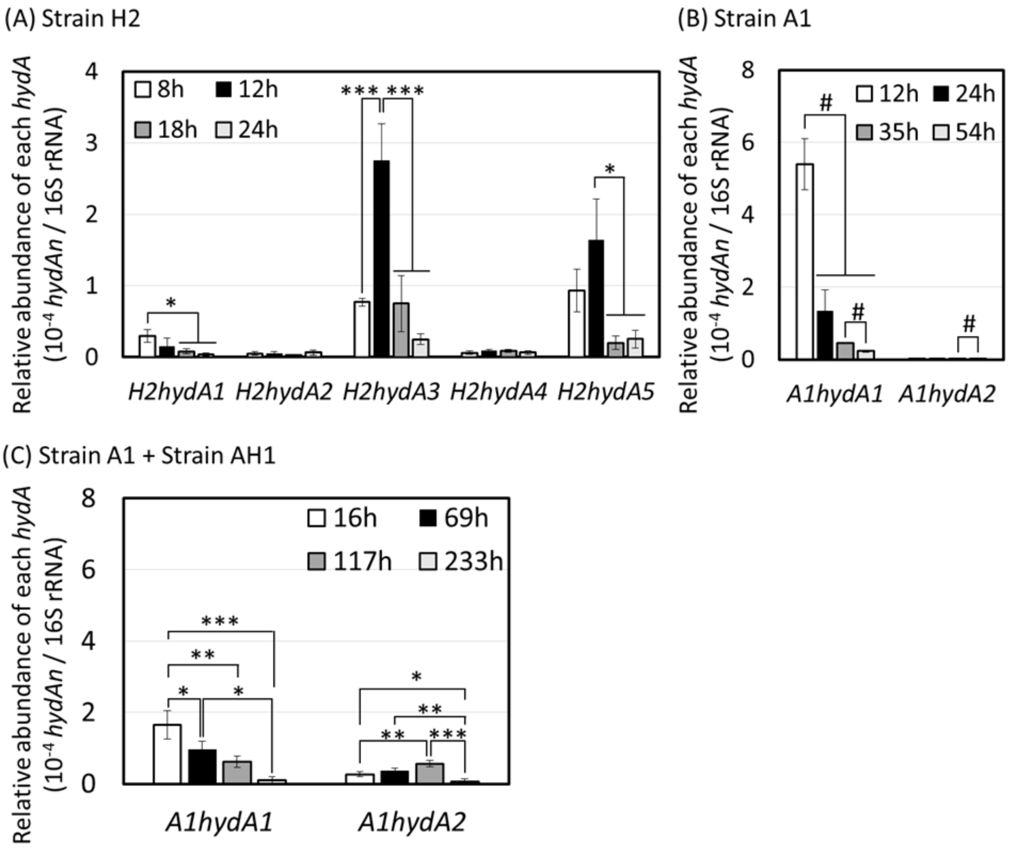

Fig. S7 Relative abundance of transcripts of each *hydA* paralog to amount of 16S rRNA of strains H2 and A1 in monoculture of (A) strain H2 and (B) strain A1, and (C) coculture of strain A1 and strain AH1. *n*h shows the sampling points (e.g, 8h means 8 hours after inoculation). *, **, and *** indicate $0.01<p<0.05$, $0.001<p<0.01$, and $p<0.001$, respectively (Tukey-Kramer test). Dunnett's T3 test was performed for (B) strain A1 because of the heteroscedasticity, and # indicates $0.01<p<0.05$ (Dunnett's T3 test). (bars = S.D., n=3)

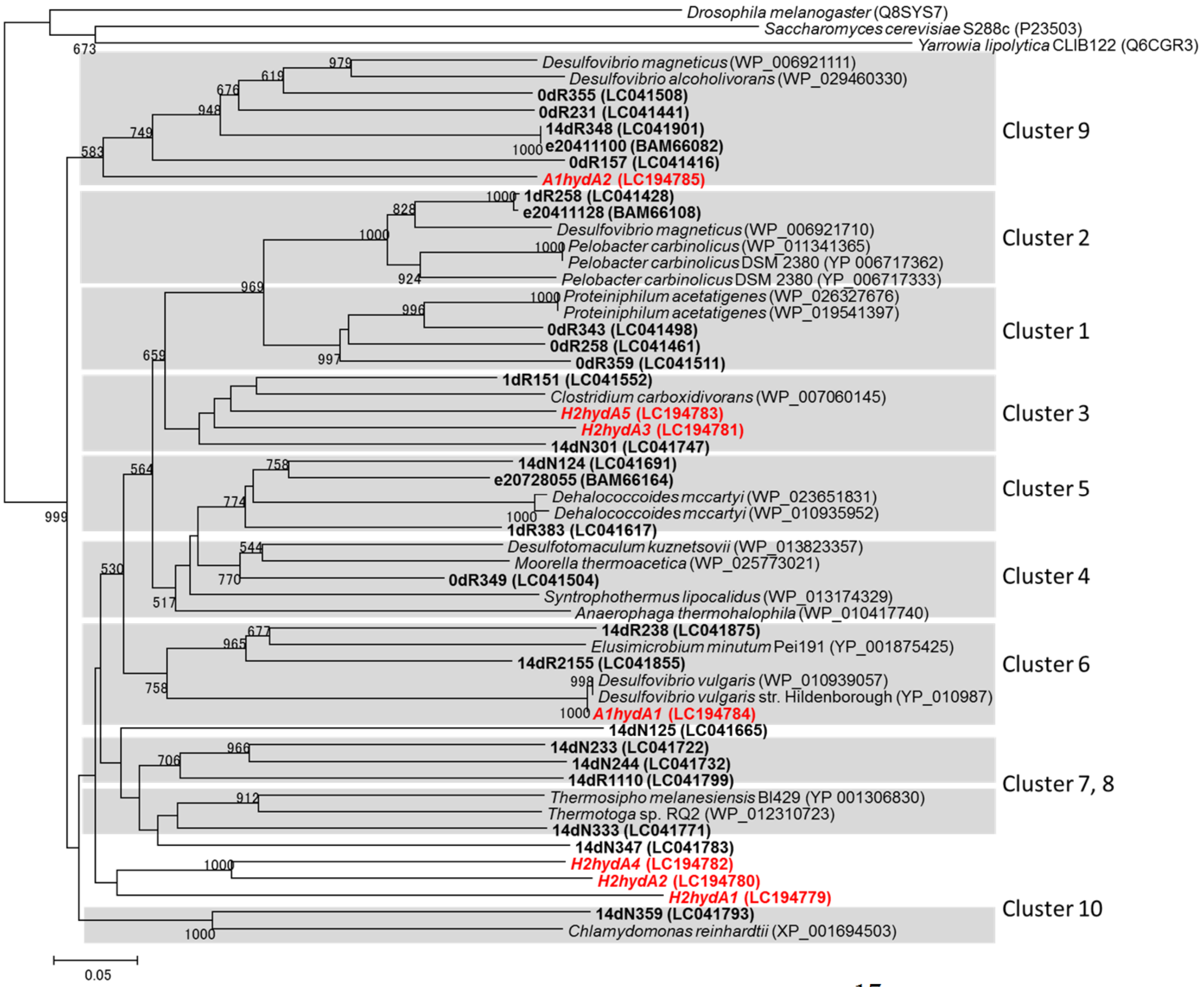

Fig. S8 Phylogenetic tree of *hydA* of *Clostridium* sp. H2 and *Desulfovibrio* sp. A1 and representative *hydA* obtained from paddy field soils (5,4). *hydA* with red color indicates the paralogs of strain H2 and A1. The numbers at the branches indicate the bootstrap values (≥50%) with 1,000 replicates. Clustering (shown in grey squares) is based on the phylogenetic relationships among the *hydA* sequences obtained in Baba *et al.*(5).

Table S1 16S rRNA, *hydA* genes and the primer sets targeting 16Sr rRNA and each *hydA* paralog.

Target		Primer[1]	Sequences (5'-3')	Accession no. of H2 or A1	Products (bp)	Reference		
						Strain	Accession no.	Length(bp)
H2	16S rRNA	27f(7)<br>1492r(7)	AGAGTTTGATCCTGGCTCAG<br>GGTTACCTTGTTACGACTT	LC194786	1463	-	-	-
	*H2hydA*1	hydA1_81f<br>hydA1_1886r	CATAAATAGCTTGTACCGCTGG<br>TCACAGTGTTTAGTGAAGAAGAGC	LC194779	1806	ATCC 638	WP_021433568	1935
	*H2hydA*2	hydA2_30f<br>hydA2_1154r	TCCAATAGAACTAGACAACCCATC<br>CCAGCTCCACCGATACATCC	LC194780	1125	ATCC 638	WP_021432477	1401
	*H2hydA*3	hydA3_145f<br>hydA3_1541r	TGCGCTTCTTGCCGTGTATG<br>GGTTGACCTGCTCCTGCTATAC	LC194781	1397	ATCC 638	WP_021434166	1773
	*H2hydA*4	hydA4_506f<br>hydA4_1814r	GTCCTTCATGGGTAAAATATGCAG<br>TCTGCATCTCTAGCAAATCTAGC	LC194782	1309	ATCC 638	WP_021431890	2061
	*H2hydA*5	hydA19299_43f<br>hydA19299_1584r	GGCCTTATGAGCTCCAGGTG<br>GCTTCATGCGGAGTTTGTAAAG	LC194783	1542	ATCC 19299	WP_021430888	1728
A1	16S rRNA	27f(7)<br>1492r(7)	AGAGTTTGATCCTGGCTCAG<br>GGTTACCTTGTTACGACTT	AB252583	1471	-	-	-
	A1hydA1	DvulhydA1_28f<br>DvulhydA1_1255r	GAGGCGCTTCTTCAGGCC<br>CGTCATGGAGCGCATCGAATATG	LC194784	1228	Hildenborough	WP_010939057	1266
	A1hydA2	DvulhydA2_22f<br>DvulhydA2_1820r	AAGGAAGTCCGGTGTGAACC<br>CAATCAGAGTCGCCCCGTAC	LC194785	1799	Hildenborough	WP_010939059	1821

[1] Primer sets for *hydA* were designed from the corresponding *hydA* sequences of reference strains (“Reference”).

Table S2 Primer sets for RT-qPCR targeting 16S rRNA and each *hydA* paralog.

Target strain	Primer	Sequences (5'-3')	Target gene	Product (bp)
H2	H216S_778f	ACTAGGTGTCGGGGGTTACC	16S rRNA	184
	H216S_961r	GAGGTCAGTGGGATGTCAAGC		
	hydA1_q_787f	TGTAAGGTCTGCTGCCCAAG	*H2hydA1*	234
	hydA1_q_1020r	GCATCATGCCCAGTTGATGC		
	hydA2_q_103f	TGCGTGCAAGCTTGTCCTAA	*H2hydA2*	122
	hydA2_q_224r	GCAACTCTAACAGCTGGAGC		
	hydA3_q_410f	GCTGTCTGTCCTACAGGAGC	*H2hydA3*	105
	hydA3_q_514r	AGCTGGAGCAACTTGAACCA		
	hydA4_q_395f	TGGAGGCTGCACTTAGAACG	*H2hydA4*	244
	hydA4_q_638r	CATCCTCCAGGGCAAGTCA		
	hydA19299_q_100f	ACCACCACCAACACATCCTC	*H2hydA5*	250
	hydA19299_q_349r	AGAGGCTGCACTTCGTACTG		
A1	A116S_1093f	TATTGCCAGTTGCTACCAGG	16S rRNA	245
	A116S_1273r	ACGCACTTTTTGGGATTGGC		
	DvulhydA1_q_693f	TCTTCTTGGTGAGGCGTTCC	*A1hydA1*	217
	DvulhydA1_q_909r	AAGGACGGCAAGGTGAGATG		
	DvulhydA2_q_1332f	GGAACTCGCCCCAGTAGAAC	*A1hydA2*	185
	DvulhydA2_q_1516r	CCATGACCTCCACGAACACA		
